# Association of the Lung Immune Prognostic Index with Immunotherapy Outcomes in Mismatch Repair Deficient Tumors

**DOI:** 10.3390/cancers13153776

**Published:** 2021-07-27

**Authors:** Edouard Auclin, Perrine Vuagnat, Cristina Smolenschi, Julien Taieb, Jorge Adeva, Laetitia Nebot-Bral, Marta Garcia de Herreros, Rosario Vidal Tocino, Federico Longo-Muñoz, Yola El Dakdouki, Patricia Martín-Romano, Lydia Gaba, Tamara Saurí, Helena Oliveres, Eduardo Castañón, Rocio Garcia-Carbonero, Benjamin Besse, Christophe Massard, Laura Mezquita, Antoine Hollebecque

**Affiliations:** 1 Gastrointestinal and Medical Oncology Department, Hôpital Européen Georges Pompidou, Université de Paris, 75015 Paris, France; edouard.auclin@aphp.fr (E.A.); jtaieb75@gmail.com (J.T.); 2 Early Drug Development Department, Institut Gustave Roussy, 94805 Villejuif, France; vuagnatperrine@gmail.com (P.V.); cristina.smolenschi@gustaveroussy.fr (C.S.); yoladakdouki@hotmail.com (Y.E.D.); Patricia.MARTIN-ROMANO@gustaveroussy.fr (P.M.-R.); Christophe.MASSARD@gustaveroussy.fr (C.M.); antoine.hollebecque@gustaveroussy.fr (A.H.); 3 Medical Oncology Department, Hospital Universitario 12 de Octubre, Imas 12, UCM, 28041 Madrid, Spain; jorge.adeva@salud.madrid.org (J.A.); rgcarbonero@gmail.com (R.G.-C.); 4 UMR9019 Genome Integrity and Cancers, Gustave Roussy Cancer Campus, 94805 Villejuif, France; laetitia.nebot-bral@gustaveroussy.fr; 5 Paris Saclay, Paris Sud University Orsay, 91400 Orsay, France; 6 Medical Oncology Department, Hospital Clinic of Barcelona, 08036 Barcelona, Spain; garciadehe@clinic.cat (M.G.d.H.); lgaba@clinic.cat (L.G.); sauri@clinic.cat (T.S.); oliveres@clinic.cat (H.O.); 7 Medical Oncology Department, Hospital Universitario de Salamanca, IBSAL, 37007 Salamanca, Spain; mrvidal@saludcastillayleon.es; 8 Medical Oncology Department, Hospital Universitario Ramon y Cajal, IRYCIS, CIBERONC, 28034 Madrid, Spain; fedelongomunoz@hotmail.com; 9 Translational Genomics and Targeted Therapies in Solid Tumors, IDIBAPS, 08036 Barcelona, Spain; 10 Department of Oncology, CUN-Madrid, 28027 Madrid, Spain; ecastanon@unav.es; 11 Medical Oncology, Institut Gustave Roussy, 94805 Villejuif, France; benjamin.besse@gustaveroussy.fr

**Keywords:** LIPI, dNLR, LDH, MSI-H, dMMR, immunotherapy, immune checkpoint inhibitors

## Abstract

**Simple Summary:**

Deficient Mismatch Repair (dMMR) is an oncogenic path accounting for around 15% of cancers. It is considered as the first predictive marker of efficacy for immune checkpoint inhibitors (ICI). However, around 39% of cases are refractory and additional biomarkers are needed. The Lung Immune Prognostic Index (LIPI) is a score reflecting the host inflammation, based on lactate deshydrogenase level, and derived neutrophils to leucocytes ratio. We aimed to assess the LIPI as a prognostic factor for ICI efficacy in patients with dMMR tumors. We found that patients with a Poor LIPI were more likely to experience disease progression, fast progression (death within the first 3 months of ICI), and have shorter overall and progression free survivals. This score is a low-cost, simple, and accessible prognostic tool in dMMR that merits further investigation in prospective studies.

**Abstract:**

**Background:** MSI-H/dMMR is considered the first predictive marker of efficacy for immune checkpoint inhibitors (ICIs). However, around 39% of cases are refractory and additional biomarkers are needed. We explored the prognostic value of pretreatment LIPI in MSI-H/dMMR patients treated with ICIs, including identification of fast-progressors. **Methods:** A multicenter retrospective study of patients with metastatic MSI-H/dMMR tumors treated with ICIs between April 2014 and May 2019 was performed. LIPI was calculated based on dNLR > 3 and LDH > upper limit of normal. LIPI groups were good (zero factors), intermediate (one factor) and poor (two factors). The primary endpoint was overall survival (OS), including the fast-progressor rate (OS < 3 months). **Results:** A total of 151 patients were analyzed, mainly female (59%), with median age 64 years, performance status (PS) 0 (42%), and sporadic dMMR status (68%). ICIs were administered as first or second-line for 59%. The most frequent tumor types were gastrointestinal (66%) and gynecologic (22%). LIPI groups were good (47%), intermediate (43%), and poor (10%). The median follow-up was 32 months. One-year OS rates were 81.0%, 67.1%, and 21.4% for good, intermediate, and poor-risk groups (*p* < 0.0001). After adjustment for tumor site, metastatic sites and PS, LIPI remained independently associated with OS (HR, poor-LIPI: 3.50, 95%CI: 1.46–8.40, *p* = 0.02. Overall, the fast-progressor rate was 16.0%, and 35.7% with poor-LIPI vs. 7.5% in the good-LIPI group (*p* = 0.02). **Conclusions:** LIPI identifies dMMR patients who do not benefit from ICI treatment, particularly fast-progressors. LIPI should be included as a stratification factor for future trials.

## 1. Introduction

Microsatellites are DNA sequences composed of short nucleotide segments which repeat sequentially. Because of their repetitive nature, these microsatellites are prone to DNA polymerase pausing and slippage during replication. In normal tissue, the mismatch repair (MMR) system usually corrects these errors [1]. However, this process may malfunction due to a constitutional or sporadic mutation in one of the repair proteins. In this case, mismatches and insertions/deletions of nucleotides accumulate in the microsatellites, resulting, first, in punctual or frameshift mutations and, second, in the activation of oncogenic genes and hypermutated phenotypes, known as microsatellite instability (MSI) or MMR deficiency (dMMR).

dMMR were initially described in colorectal cancer (CRC), reported to be involved in around 15% of tumors and could be related to Lynch syndrome, an autosomal dominant cancer-predisposition syndrome caused by mutations in MMR strains [2]. However, MSI can also be observed in sporadic CRC, which is more likely related to the epigenetic inactivation of the MLH1 gene expression promoter. MSI has also been observed in other tumor types (1% to 30% depending on the histological type), with endometrial carcinoma and gastric cancer being the most common [3,4].

MSI-H and dMMR, both early conditions of hypermutability, increase the generation of neoantigens leading to increased tumor immunogenicity and high tumor mutational burden [5], suggesting that immunotherapy could be an interesting approach in patients harboring these mutations. Research led to dMMR receiving FDA-approval as the first agnostic tumor-related predictive biomarker for PD-1 inhibitors. In a cohort of patients with MSI tumors, pembrolizumab demonstrated response in between 40–70% of patients vs. none in microsatellite stable tumors [6]. Following this trial, other studies have demonstrated similar findings with other PD-1 inhibitors [7,8,9,10]. 

Despite the impressive data reported, not all patients with dMMR tumors respond to PD-1 inhibitors, and there is a need for additional biomarkers for immune checkpoint inhibitors (ICIs). Host-related biomarkers are gaining importance in immune-oncology, providing additional information from the patient (host) that can be integrated into immunotherapy outcome prediction. Among them, the lung immune prognostic index (LIPI), based on two inflammatory parameters (derived neutrophil to leucocytes ratio [dNLR] calculated as neutrophil count/[leucocytes count—neutrophils count], and lactate dehydrogenase [LDH]) correlated strongly with immunotherapy outcomes in large cohorts of patients with non-small cell lung cancer, and in other tumor types such as renal and head and neck cancers [11,12,13,14,15].

In this study, we aimed to assess the prognostic value of LIPI in a large multicenter cohort of patients treated with immunotherapy for dMMR tumors, and whether LIPI can identify fast progressors who are undergoing immunotherapy.

## 2. Materials and Methods

### 2.1. Study Design and Patients

This is a retrospective multicenter study of patients with advanced solid tumors with dMMR status, treated with anti-PD-1 or PD-L1 ICIs (atezolizumab, avelumab, nivolumab +/− ipilimumab, pembrolizumab, and other phase I drugs) in a variety of settings including routine clinical care, expanded access programs, and clinical trials, between April 2014 and May 2020 across seven European tertiary referral hospitals in France and Spain. We collected baseline clinical, pathological, and biological data prior to immunotherapy, including leucocyte and neutrophil counts, LDH, and albumin. Radiological assessment was performed every 8–12 weeks according to local practice and evaluated per Response Evaluation Criteria in Solid Tumors v1.1 (RECIST). The study was approved by the Institutional Review Board of the Institut Gustave Roussy on 18th March 2021 (Registration Number 2021-26). Informed consent was not required for this retrospective study.

### 2.2. Lung Immune Prognostic Index

The LIPI was calculated based on dNLR > 3 (one point) and LDH > upper limit of normal (ULN) (one point), as previously reported [11]. Patients with no points were classified in the good group, patients with 1 point (dNLR > 3 or LDH > ULN) in the intermediate group, and those with 2 points (dNLR > 3 and LDH > ULN) in the poor group.

### 2.3. MSI-H/dMMR Status

MMR status was analyzed according to the techniques used in each center, including immunohistochemistry, polymerase chain reaction (PCR), next-generation, or whole exome sequencing.

### 2.4. Statistical Analysis

Median (IQR) values and proportions (percentage) were used for continuous and categorical variables, respectively. Median and proportions were compared using the Wilcoxon–Mann–Whitney test and the chi^2^ test (or Fisher’s exact test, if appropriate), respectively. The primary endpoint was overall survival (OS) defined as the time between the start of ICI treatment and death from any cause. The other primary endpoint was the fast progressors rate (FPR), defined as an OS < 3 months. Secondary endpoints were progression-free survival (PFS), ORR, and disease control rate (DCR). PFS was defined as the time between ICI start and progressive disease (PD) or death, whichever occurred first. ORR was defined as the sum of complete and partial responses according to RECIST 1.1. DCR was defined as the sum of complete and partial responses and stable disease according to RECIST 1.1. OS and PFS were estimated using the Kaplan Meier method, and groups were compared with the log-rank test. Follow-up was calculated using the reverse Kaplan Meier method. The association of demographic, clinical, and biological factors with survival was assessed with univariate and multivariate Cox proportional-hazard models, providing a hazard ratio (HR) and its 95% confidence interval (CI). The association between LIPI and FPR ORR and DCR was evaluated with logistic regression, providing an odds ratio (OR) and its 95%CI. All analyses were performed using R software version 2.15.2 (R Development Core Team, Vienna, Austria). *p* values < 0.05 were considered statistically significant, and all tests were two-sided.

## 3. Results

### 3.1. Study Population

A total of 151 patients were included with a median follow-up of 32.1 months (95%CI 24.8–36.3). 

The main baseline characteristics are summarized in Table 1. The most common tumor types were gastrointestinal (65.6%; 60.6% CRC, 39.4% others) followed by gynecologic tumors (21.8%) (Figure S1). In the 146 patients with the available data, dMMR status was diagnosed by PCR alone in 5 patients (3.4%), immunohistochemistry and PCR in 135 patients (92.5%), with next-generation or whole exome sequencing (alone or in combination with the other techniques) in 11 patients (7.5%). dMMR status was associated with Lynch syndrome in 40 (32.0%) patients (80.0% CRC, 5.0% gynecologic, 15.0% other). 

Median OS was not reached (NR) in the overall population, (95%CI: 23.4 to NR), and the 1-year OS rate was 69.3% (95%CI: 62.0 to 77.6). Median PFS was 10.5 months (95%CI: 7.1 to 35.1) and the 1-year PFS rate was 47.8% (95%CI: 40.1 to 56.9) (Table 2).

### 3.2. LIPI in dMMR Tumors

Pretreatment median dNLR was 2.29 (interquartile range, IQR: 1.61–3.09) and was >3 in 25.8% of patients. Pretreatment median LDH was 218 IU/L (IQR: 188.5–313) and was >ULN in 38.5%. Considering both parameters, LIPI was evaluable in 143 patients, and classified the population into three prognostic groups: good (*n* = 67, 46.9%), intermediate (*n* = 62, 43.3%) and poor (*n* = 14, 9.8%) (Figure S2). The baseline characteristics of the population by LIPI group (*n* = 143) are summarized in Tables S1 and S2. The presence of brain metastasis, high number of metastatic sites (>2), poor PS and hypoalbuminemia at ICI start were associated with poor LIPI.

### 3.3. LIPI Is Associated with ICI Survival Outcomes in MSI-H Tumors 

**Figure 1:**
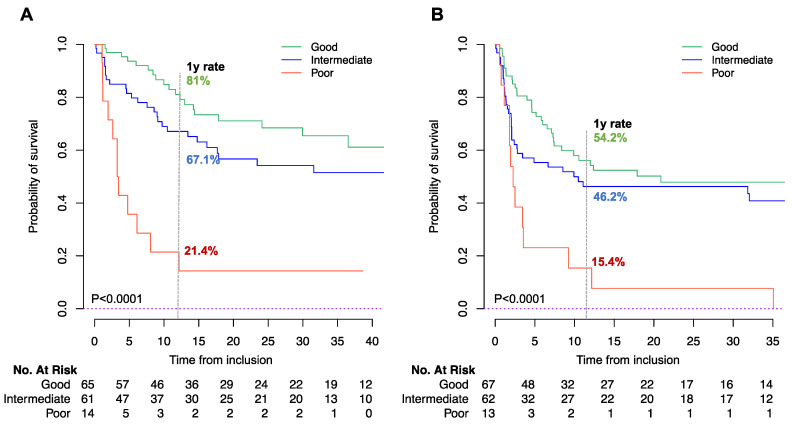
Kaplan Meier curve for OS (**A**) and PFS (**B**) according to LIPI score. Log Rank *p* < 0.0001 for both endpoints. The numbers at risk differ between OS and PFS, due to missing data in OS/PFS status or duration.

LIPI was associated with both OS and PFS (*p* < 0.0001). Median OS was NR (95%CI 36.5 to NR), NR (95%CI 16.2 to NR), and 3.3 months (95%CI 2.6 to NR) for the good, intermediate, and poor LIPI groups, respectively (*p* < 0.001) (Figure 1A, Table 2). The one-year OS rates for good, intermediate, and poor-LIPI groups were 81.0% (95%CI 71.5 to 91.9), 67.1% (95%CI 56.0 to 80.5), and 21.4% (95%CI 7.9 to 58.4), respectively (*p* < 0.001). 

Similarly, median PFS was 20.9 months (95%CI 8.4 to NR), 9.9 months (95%CI 2.8 to NR), and 2.3 months (95%CI 1.8 to NR) in the good, intermediate, and poor-LIPI groups, respectively (*p* < 0.0001; Table 2). The one-year PFS rates for good, intermediate, and poor-LIPI groups were 54.2% (95%CI 43.1 to 68.2), 46.2% (95%CI 35.1 to 61.0), and 15.4% (95%CI 4.3 to 55.0), respectively (*p* < 0.0001), Figure 1B.

In the multivariate analysis, including tumor location, number of metastasis before ICI, ECOG PS, and albumin levels, LIPI was an independent factor for OS (HR for intermediate, 1.43 [95%CI 0.75 to 2.74]; HR for poor, 3.50 [95%CI 1.46 to 8.40], *p* = 0.03). In terms of PFS, the HRs for the intermediate and poor groups were 1.09 (95%CI 0.65 to 1.82) and 2.41 (95%CI 1.12 to 5.19), respectively (*p* = 0.07) (Table 3). The c-index of LIPI for OS and PFS prediction are reported in Table S3.

### 3.4. LIPI Is Associated with Tumor Response under ICI in dMMR Tumors

**Figure 2:**
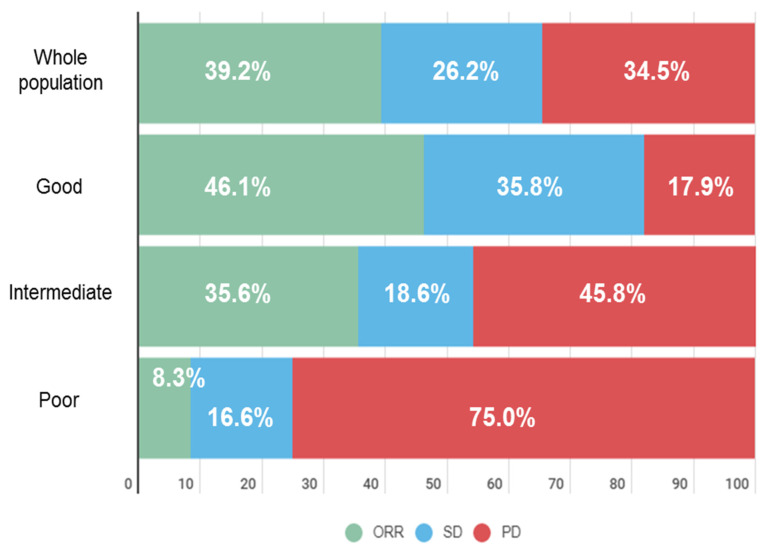
Best response according to LIPI group. ORR: objective response rate, SD: stable disease, PD: progression disease.

We also studied the impact of LIPI on response outcomes. The ORR in the overall population was 39.2%. According to LIPI group, the ORR was 46.2% in the good group, 35.6% in the intermediate, and 8.3% in the poor group (*p* = 0.03) (Table 2, Figure 2). Patients in the poor-LIPI group had a higher risk of experiencing nonresponse compared with the good-LIPI group, with an OR 9.43 (95%CI 1.15 to 77.27, *p* = 0.04) (Table 4). Similarly, PD as best response (absence of DCR), was significantly associated with the poor-LIPI group.

### 3.5. Fast Progressors Rate

We also evaluated the FPR in our dMMR population. Overall, the FPR was 16.0% (*n* = 24/150) (Table 2). The distribution of fast progressors was not different among the different tumor types. When considered with reference to the LIPI group, the FPR was significantly higher in the poor -LIPI group, with 35.7% of patients (*n* = 5/14), compared with the intermediate group with 18.0% (*n* = 11/62) and the good-LIPI group with 7.5% (*n* = 5/67), *p* = 0.02 (Table 2). Additionally, the poor group had a significantly higher risk of experiencing fast progression compared with the good-LIPI group, with an OR of 6.89 (95%CI 1.66 to 28.59, *p* = 0.01) (Table 4).

## 4. Discussion

In this study, we have shown for the first time the impact of host-related biomarkers, represented by circulating inflammatory parameters combined as the LIPI score (dNLR and LDH), on ICI outcomes in a large cohort of patients with advanced dMMR solid tumors, widely considered a favorable population for ICI. In this study, LIPI was an independent prognostic factor for OS, with the poor-LIPI group being associated with worse immunotherapy outcomes, suggesting that host-related biomarkers are also relevant for this population. This poor-LIPI group comprised a subset of patients (9.8% of the patients) with no clear benefit for immunotherapy, despite of their dMMR status, with a median OS of 3.3 months, median PFS of 2.3 months, and a 35.7% rate of fast-progressors.

Interestingly, we observed in our population, composed primarily of gastrointestinal and gynecologic tumors, similar data as previously reported in other tumor types, notably NSCLC [11,12,13,14,15], suggesting that these parameters reflect the host immune context regardless of the tumor type and other tumor-related biomarkers, such as dMMR. Of note in these previous studies, median OS for the evaluated poor-LIPI subgroups ranged from 2.6 to 5.0 months, and median PFS ranged from 1.2 to 2.3 months [11,12,13,14,15,16]. 

Inflammation and activation of the innate immune system is a well-known resistance pathway for ICIs, promoting tumor growth and dissemination [17,18,19]. Among the innate immune cells, neutrophils are one of the major actors. As a neutrophil-based ratio, LIPI is thus a good indicator of the circulating inflammatory status of patients before receiving ICI therapy. Systemic inflammation is capable of inducing IDO (indoleamine 2,3-dioxygenase) and also turning immune infiltration towards immunotolerance [20]. IDO plays an immunosuppressive role, preventing perpetual inflammation. IDO expression in MSI tumors is heterogeneous [21]. High IDO expression induced by systemic inflammation could explain the differences in response to ICIs, even in MSI patients. It has also been described that MSH3 mutations, which result in the EMAST phenotype (elevated microsatellite alterations at selected tetranucleotide repeats), could lead to chronic inflammation mediated by IL-6 and TNF-alpha [22]. These EMAST patients had shorter survival and an aggressive tumor phenotype. The good prognostic value of the LIPI score in the MSI population is explained by its capacity to reflect the patient’s systemic inflammation.

dMMR is a validated, FDA-approved, tumor-based predictive biomarker of response to ICI, with impressive data in terms of response (40% to 55% in CRC with dMMR, and 34% to 71% in non-CRC tumors) [7,8,9,10]. Despite these encouraging ORRs, PD as best response can represent 12% to 61% of this population, depending on the histological subtype, and no biomarkers are available to screen these patients in order to address this [6]. In our study, we observed 32.6% of patients with refractory disease having PD as best response, however this increased significantly in the poor-LIPI group, reaching 75%. This suggested that LIPI could be a useful tool to better identify true responders and, even more relevantly, to identify populations unlikely to respond to immunotherapy, providing additional information on outcome prediction, compared to the restricted vision, when using only tumor-based biomarkers. Similarly, LIPI, as for other host-related biomarkers, has also been explored in ≥50% PD-L1 NSCLC, another favorable population for immunotherapy [23]. In a cohort of 930 patients treated with ICIs, LIPI was an independent prognostic marker regardless of PD-L1 expression. Both this and our study have highlighted the concept that LIPI can provide additional relevant information to already-known tumor-based biomarkers (e.g., PD-L1, dMMR), and could be explored in combination with these well-established biomarkers in clinical trials. The predictive values of LIPI on ICI benefit have already been investigated in NSCLC [24,25].

In our study, we defined fast progressors as patients with an OS < 3 months after ICI start. This is one of the aggressive progression patterns described in cancer patients under immunotherapy [23,26]. Although the immunological mechanisms have not been well established and this phenomenon remains controversial, some patients experience rapid and aggressive progression and previous reports highlighted inflammation as a key mechanism for the aggressive patterns [26,27]. In our study, we described 16% of our population as fast progressors, however this proportion was notably higher (35%) in the poor-LIPI population. This could be the first clinical evidence of the link between circulating inflammatory status and refractory disease under immunotherapy. In this context, we observed that single agent ICIs are not able to overcome this primary resistance, even in a good-responder population (dMMR). In the future, LIPI could serve as a tool for clinicians to select the best treatment strategy, as is being explored in NSCLC [23,24,25].

Our study has a number of limitations directly related to its retrospective nature, notably missing clinical data (for example other possible interesting inflammatory biomarkers such a C-Reactive Protein). Secondly, patients were treated with various immunotherapy drugs, which can lead to heterogeneity in terms of efficacy; nevertheless, our cohort is representative of the dMMR population, with outcome data for response and survival consistent with previous data reported in the literature. Finally, the lack of other treatment cohorts with combination therapies or only chemotherapy as a comparison limited our exploration of the potential predictive rather than prognostic role of LIPI in this dMMR cohort. 

Nonetheless, this study represents the first proof of concept that the patient’s host-immune context can play an important role in dMMR patients receiving immunotherapy. We demonstrated, in the largest multicenter cohort of patients with dMMR tumors reported to date, that LIPI can offer useful information on outcome prediction to the current exclusive context of tumor-based biomarkers. The integration of these host-related biomarkers with tumor-based biomarkers will improve identification of relevant data in the decision-making process for selecting the best therapeutic strategy in cancer patients.

## 5. Conclusions

The LIPI, based on pretreatment dNLR and LDH, is associated with ICI outcomes in the MSI-H/d-MMR population, with LIPI demonstrated to be an independent factor for OS. LIPI can identify the population with higher risk of progression or death under ICIs, the poor-LIPI group with high-dNLR/high-LDH. This score is a low-cost, simple, and accessible prognostic tool in dMMR that merits further investigation in prospective studies.

## Figures and Tables

**Table 1:** Clinical, pathological and biological characteristics of the population.

Variable		All Patients (N = 151)
Age	Median (IQR)	64 (51.5-70.5)
	>65	64 (43.0%)
	Missing	2
Gender	female	89 (58.9%)
	male	62 (41.1%)
Primary tumor site	gastrointestinal	99 (65.6%)
	gynecologic	33 (21.8%)
	other	19 (12.6%)
Lynch syndrome	yes	40 (32.0%)
	missing	26
Line of ICI start	median (IQR)	2 (2–3)
	>2	62 (41.3%)
	missing	1
Number of metastasis at ICI start	>2	34 (23.6%)
	missing	7
Metastasis sites	lung metastasis	31 (20.5%)
	bone metastasis	13 (8.6%)
	liver metastasis	47 (31.1%)
	brain metastasis	7 (4.6%)
Type of ICI antibody	PD-1	109 (72.2%)
	PD-L1	42 (27.8%)
Monotherapy or combination	combination	20 (13.3%)
	monotherapy	131 (86.7%)
Performance status at ICI start	0	58 (42.3%)
	≥1	79 (57.7%)
	missing	14
dNLR	>3	39 (25.8%)
LDH	high	55 (38.5%)
	missing	8
Albumin (g/L)	≤35	64 (43.5%)
	missing	4

ICI: immune checkpoint inhibitors, dNLR: derived neutrophils to leukocytes ratio, LDH: lactate dehydrogenase, LIPI: lung immune prognostic index.

**Table 2:** Median survival and response endpoints according to the LIPI score.

Variable		All Patients (N = 151)	LIPI Good Prognostic Group (N = 67)	LIPI Intermediate Prognostic Group (N = 62)	LIPI Poor Prognostic Group (N = 14)	*p*
Median (95%CI)	OS	NR (23.4 to NR)	NR (36.5 to NR)	NR (16.2 to NR)	3.3 (2.6 to NR)	<0.001
	PFS	10.5 (7.1 to 35.1)	20.9 (8.4 to NR)	9.9 (2.8 to NR)	2.3 (1.8 to NR)	<0.001
Fast progressors rate	yes	24 (16.0%)	5 (7.5%)	11 (18.0%)	5 (35.7%)	0.02
	no	126 (84.0%)	62 (92.5%)	50 (82.0%)	9 (64.3%)	
	missing	1	0	1	0	
ORR	no	87 (60.8%)	35 (53.8%)	38 (64.4%)	11 (91.7%)	0.03
	yes	56 (39.2%)	30 (46.2%)	21 (35.6%)	1 (8.3%)	
	missing	8	2	3	2	
DCR	no	47 (32.6%)	10 (15.2%)	26 (44.1%)	9 (75.0%)	<0.001
	yes	97 (67.4%)	56 (84.8%)	33 (55.9%)	3 (25.0%)	
	missing	7	1	3	2	

95%CI: 95% confidence interval, ORR: objective response rate, DCR: disease control rate.

**Table 3:** Multivariate Cox model analysis for OS and PFS in the population.

Variable		OS N = 125, n Events = 49			PFS N = 124, n Events = 71		
		HR	95%CI	*p*	HR	95%CI	*p*
**Tumor site**	gastrointestinal	1			1		
	gynecologic	1.65	0.82 to 3.29	0.03	1.53	0.84 to 2.81	0.0002
	other	2.59	1.25 to 5.35		4.08	2.08 to 8.01	
**N metastatic sites at ICI start**	>2	1.99	1.06 to 3.70	0.03	1.06	0.61 to 1.85	0.84
**Performance status**	0						
	≥1	2.11	1.05 to 4.24	0.04	1.91	1.10 to 3.31	0.02
**Albumin (g/L)**	>35	0.82	0.45 to 1.50	0.51	0.96	0.58 to 1.59	0.87
**LIPI**	good	1			1		
	tntermediate	1.43	0.75 to 2.74	0.02	1.09	0.65 to 1.82	0.07
	poor	3.50	1.46 to 8.40		2.41	1.12 to 5.19	

OS: overall survival, PFS: progression-free survival, HR: hazard ratio, 95%CI: 95% confidence interval, ICI: immune checkpoint inhibitors, LIPI: lung immune prognostic index.

**Table 4:** Univariate logistic regression for response endpoints according to LIPI score.

Variable	ORR		DCR		Fast Progressors	
	OR (95%CI)	*p*	OR (95%CI)	*p*	OR (95%CI)	*p*
LIPI						
Good	Ref		Ref		Ref	
Intermediate	1.55 (0.75–3.19)	0.23	4.41 (1.89–10.29)	0.01	2.73 (0.89–8.37)	0.08
Poor	9.43 (1.15–77.27)	0.04	16.8 (3.86–73.05)	<0.0001	6.89 (1.66–28.59)	0.01

ORR: objective response rate, DCR: disease control rate, OR: odds ratio, Ref: reference.

## Data Availability

Contact the corresponding author.

## Supplementary Materials

The following are available online at https://www.mdpi.com/article/10.3390/cancers13153776/s1, Figure S1: distribution of tumor types in the study population (*n* = 151), Figure S2: distribution of the LIPI groups in the study population, Table S1: clinical, pathological and biological characteristics of the population according to LIPI group (*n* = 143), Table S2: detailed tumor types according to LIPI group (*n* = 143), Table S3: LIPI c-index for OS and PFS prediction.

## Abbreviations

dMMR	deficient mismatch repair
dNLR	derived neutrophils/leucocytes ratio
LIPI	lung immune prognostic index
MSI-H	microsatellites instable high
OS	overall survival
PFS	progression free survival
