# Supplementary material for: Association of the Lung Immune Prognostic Index with Immunotherapy Outcomes in Mismatch Repair Deficient Tumors
Source: Cancers (Basel). 2021 Jul 27;13(15):3776. doi: 10.3390/cancers13153776 (PMC8345164; doi:10.3390/cancers13153776)
Supplement: Supplementary file 1 [file cancers-13-03776-s001.zip › cancers-1271103-supplementary.pdf]

cancers

MDPI

Article

Association of the Lung Immune Prognostic Index with Immunotherapy Outcomes in Mismatch Repair Deficient Tumors

Edouard Auclin, Perrine Vuagnat, Cristina Smolenschi, Julien Taieb, Jorge Adeva, Laetitia Nebot-Bral, Marta Garcia de Herreros, Rosario Vidal Tocino, Federico Longo-Muñoz, Yola El Dakdouki, Patricia Martín-Romano, Lydia Gaba, Tamara Saurí, Helena Oliveres, Eduardo Castañón, Rocío Garcia-Carbonero, Benjamin Besse, Christophe Massard, Laura Mezquita and Antoine Hollebecque

Table S1. Clinical, pathological and biological characteristics of the population according to LIPI group (n = 143).

		LIPI Good prognostic group (N = 67)	LIPI Intermediate prognostic group (N = 62)	LIPI Poor prognostic group (N = 14)	*p* value
Age (years)	Median (IQR)	65 (52.5;69.5)	59.5 (49;70)	67 (62.5;71.5)	0.26
	> 65	30 (46.2%)	23 (37.1%)	8 (57.1%)	0.32
	Missing	2	0	0	
Gender	Female	35 (52.2%)	39 (62.9%)	8 (57.1%)	0.47
	Male	32 (47.8%)	23 (37.1%)	6 (42.9%)	
Primary tumor site	Gastrointestinal	53 (79.1%)	37 (59.7%)	6 (42.8%)	0.02
	Gynecologic	9 (13.4%)	15 (24.2%)	4 (28.6%)	
	Other	5 (7.5%)	10 (16.1%)	4 (28.6%)	
Lynch syndrome	Yes	20 (38.5%)	16 (29.6%)	3 (27.3%)	0.63
	Missing	15	8	3	
Synchronous metastasis	Yes	23 (34.8%)	26 (41.9%)	5 (35.7%)	0.70
	Missing	1	0	0	
Line of ICI start	Median (IQR)	2 (2;3)	2 (2;3)	2 (2;3)	0.91
	> 2	31 (46.3%)	25 (40.3%)	4 (30.8%)	0.55
	Missing	0	0	1	
N metastasis at ICI start					
	> 2	11 (17.5%)	16 (26.7%)	6 (42.9%)	0.11
	Missing	4	2	0	
Lung metastasis	Yes	9 (13.4%)	14 (22.6%)	6 (42.9%)	0.04
Bone metastasis	Yes	2 (3.0%)	6 (9.7%)	4 (28.6%)	0.01
Liver metastasis	Yes	17 (25.4%)	23 (37.1%)	7 (50.0%)	0.13
Brain metastasis	Yes	0 (0%)	5 (8.1%)	2 (14.3%)	0.001
Type of ICI anti body	PD-1	43 (64.2%)	50 (80.6%)	9 (64.3%)	0.12
	PD-L1	24 (35.8%)	12 (19.4%)	5 (35.7%)	
Monotherapy or combination	Combination	14 (20.9%)	4 (6.5%)	1 (7.1%)	0.05
	Monotherapy	53 (79.1%)	58 (93.5%)	13 (92.9%)	
Performance status at ICI start	0	33 (55.9%)	23 (38.3%)	1 (8.3%)	0.004

	≥1	26 (44.01%)	37 (61.7%)	11 (91.7%)	
	Missing	8	2	2	
dNLR	> 3	0 (0%)	21 (33.9%)	14 (100%)	<0.0001
LDH	High	0 (0%)	41 (66.1%)	14 (100%)	<0.001
	Missing	0	0	0	
Albumin (g/L)	≤ 35	18 (26.9%)	33 (54.1%)	10 (71.4%)	0.001
	Missing	0	1	0	

ICI: immune checkpoint inhibitors, dNLR: derived neutrophils to leukocytes ratio, LDH: lactate dehydrogenase, LIPI: lung immune prognostic index.

Table S2. detailed tumor types according to LIPI group (n = 143).

Tumor site	Whole sample (N = 151)	Good (N = 67)	Intermediate (N = 62)	Poor (N = 14)
Central Nervous System	8 (5.3%)	2 (2.99%)	6 (9.68%)	0 (0%)
Colon	66 (43.71%)	35 (52.24%)	23 (37.1%)	5 (35.71%)
Gynecological	32 (21.19%)	9 (13.43%)	15 (24.19%)	3 (21.43%)
Other gastrointestinal	23 (15.23%)	14 (20.9%)	9 (14.52%)	0 (0%)
Other non responsive	11 (7.28%)	2 (2.99%)	4 (6.45%)	5 (35.71%)
Pancreas and biliary tract	11 (7.28%)	5 (7.46%)	5 (8.06%)	1 (7.14%)

Table S3. LIPI c-index for OS and PFS prediction.

	OS	PFS
Univariate analysis	0.65	0.59
Multivariate analysis	0.74	0.66

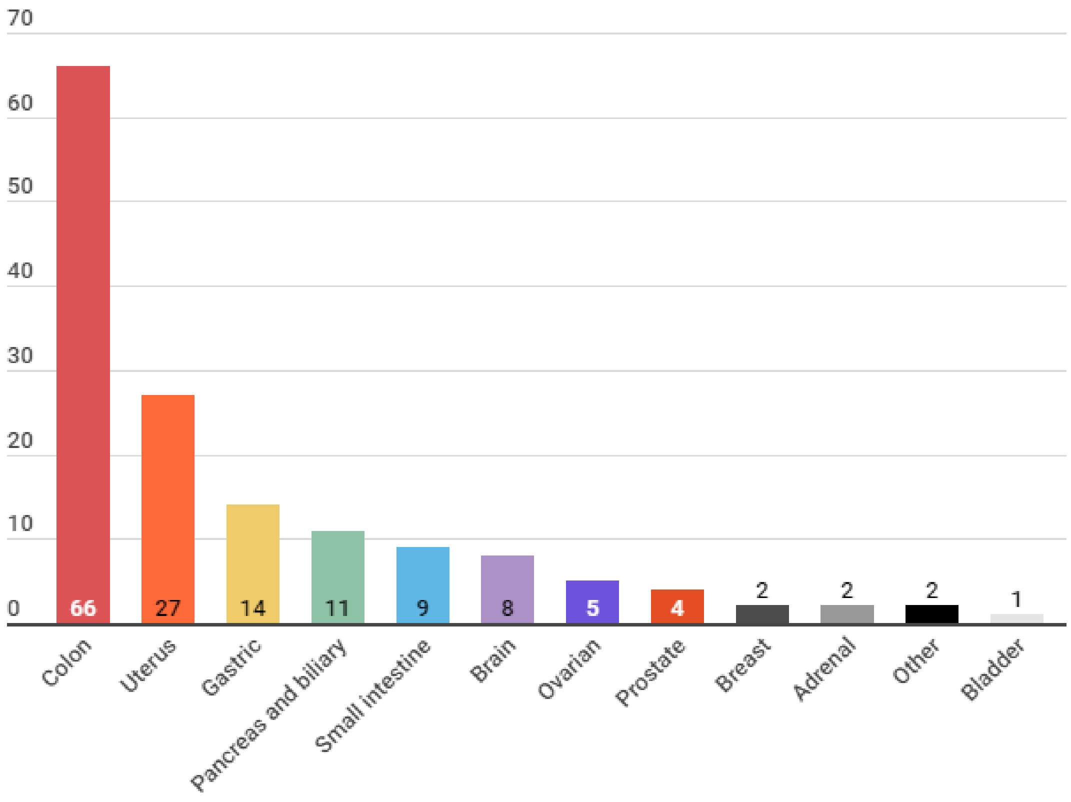

Figure S1. Distribution of tumor types in the study population (n = 151).

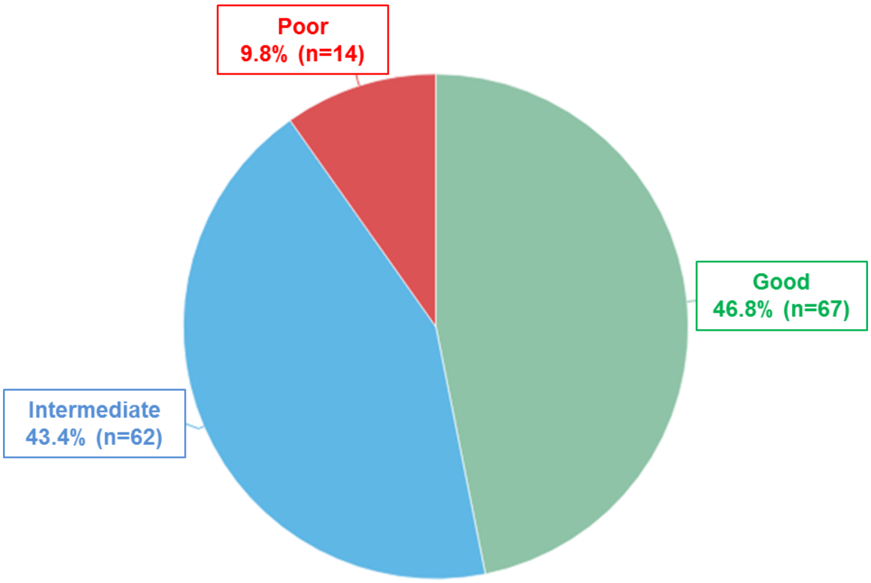

Figure S2. Distribution of the LIPI groups in the study population.
